# Efficacy of REAC Neurobiological Optimization Treatments in Post-Polio Syndrome: A Manual Muscle Testing Evaluation

**DOI:** 10.3390/jpm14101018

**Published:** 2024-09-24

**Authors:** Monalisa Pereira Motta, Acary Souza Bulle Oliveira, Jeyce Adrielly André Nogueira, Alcione Aparecida Vieira de Souza Moscardi, Vanessa Manchim Favaro, Amanda Orasmo Simcsik, Chiara Rinaldi, Vania Fontani, Salvatore Rinaldi

**Affiliations:** 1 Division of Neuromuscular Diseases, Department of Neurology and Neurosurgery, Federal University of São Paulo, Sao Paulo 04021-001, Brazil; monalisa.motta@gmail.com (M.P.M.); acary.bulle@unifesp.br (A.S.B.O.); jaanogueira21@unifesp.br (J.A.A.N.); v.favaro@unifesp.br (V.M.F.); amanda.simcsik11@gmail.com (A.O.S.); 2 Department of Preventive Medicine, Federal University of São Paulo, Sao Paulo 01000-000, Brazil; moscardi.alcione@unifesp.br; 3 Department of Adaptive Neuro Psycho Physio Pathology and Neuro Psycho Physical Optimization, Rinaldi Fontani Institute, 50144 Florence, Italyvfontani@irf.it (V.F.); 4 Department of Regenerative Medicine, Rinaldi Fontani Institute, 50144 Florence, Italy; 5 Research Department, Rinaldi Fontani Institute, 50144 Florence, Italy

**Keywords:** post-polio syndrome, REAC, neurobiological modulation, neurostimulation, manual muscle testing, motor disorders

## Abstract

**Background:** This study evaluated the effectiveness of radio electric asymmetric conveyer (REAC) neurobiological optimization treatments on muscle strength (MS) in individuals with post-polio syndrome (PPS), a condition causing new muscle weakness in polio survivors. Traditional treatments focus on symptom management, whereas REAC technology uses radio electric symmetric conveyed fields to modulate neurotransmission and cellular function. **Methods:** This open-label study involved 17 PPS patients who maintained their existing medications. The participants underwent four REAC treatment protocols: neuro-postural optimization (NPO), neuro-psycho-physical optimization (NPPO), neuro-psycho-physical optimization—cervical brachial (NPPO-CB), and neuromuscular optimization (NMO). MS was assessed using manual muscular tests (MMT) before and after each protocol. **Results:** A statistical analysis via repeated measures ANOVA showed significant MS improvements, particularly in the proximal muscles of the left lower limb (LLL), distal muscles of both lower limbs (LLs), and distal muscles of the left upper limb. The LLL, the most severely affected limb at this study’s start, exhibited the greatest improvement. **Conclusions:** These results suggest REAC treatments could enhance MS in PPS patients, potentially reorganizing motor patterns and reducing functional overload on less affected limbs.

## 1. Introduction

Post-polio syndrome (PPS) is a condition that can affect polio survivors decades after they recover from their initial infection [1]. It manifests with symptoms such as new and progressive muscle weakness, joint and muscle pain [2], fatigue [3], muscle atrophy [3], breathing or swallowing problems, and sleep disorders [4].

New neuromuscular symptoms have been observed to manifest with greater prevalence in previously affected limbs and in patients exhibiting greater residual weakness. These symptoms tend to emerge gradually over time, though they may become manifest suddenly in cases with periods of inactivity, injury, surgery, or intense physical exercise.

The etiology of PPS remains poorly understood. Among the numerous hypotheses, the most widely accepted is that PPS is caused by the overuse of surviving motor neurons over the years. This results in the disintegration of terminal axons and an intense metabolic demand.

The diagnosis of post-polio syndrome (PPS) is generally established through a process of exclusion, where other potential causes of the observed symptoms are systematically ruled out. Currently, there are no specific diagnostic tests available for PPS, so diagnosis relies on clinical criteria. At the Neuro-Muscular Diseases Sector of the Federal University of São Paulo (UNIFESP), the diagnosis is made according to well-defined criteria.

The diagnostic criteria require a history of paralytic poliomyelitis with documented evidence of motor neuron loss. This includes a confirmed history of acute paralytic disease, residual muscle weakness, and atrophy observed during neurological examination, as well as signs of denervation on electromyography. Following an acute episode of poliomyelitis, there must be either partial or complete neurological and functional recovery. Additionally, the patient must have experienced a minimum period of 15 years of neurological and functional stability after the initial recovery.

PPS is characterized by the gradual or sudden onset of new muscle weakness, with or without accompanying symptoms. Importantly, other medical conditions that could explain the symptoms must be excluded. Furthermore, the symptoms should persist for at least one year, illustrating the chronic nature of the condition.

New muscle weakness is a highly significant symptom and can be employed as a diagnostic criterion. It is typically asymmetrical and may occur in muscles previously affected by poliomyelitis, as well as in uninvolved muscles. It can be permanent or transient. It usually progresses slowly but sometimes develops in a subacute manner or progresses incrementally. Transient weakness is probably a manifestation of muscular fatigue.

In individuals who have survived poliomyelitis, the metabolic cost of walking or carrying out daily activities is significantly elevated. This is because the intensity of muscle contraction required to perform daily activities exceeds the maximum capacity of the muscles. This can potentially lead to a progressive reduction in working capacity and a feeling of fatigue that is not typical for them.

New muscle weakness is a constant and worsening symptom associated with the loss of pre-existing functional motor skills.

There is no specific cure for post-polio syndrome (PPS). Given its impact on polio survivors, investigating relevant treatments is crucial. To this end, we explored the potential of neurobiological modulation using radio electric asymmetric conveyer (REAC) technology, employing four specific treatment protocols: neuro-postural optimization (NPO) for initial and long-lasting functional reorganization of neuromotor strategies at the brain level [5]; neuro-psycho-physical optimization (NPPO) and neuro-psycho-physical optimization—cervical brachial (NPPO-CB), respectively, aimed at improving overall relational neuro-psycho-physical functions at the cortical level (NNPO) and at the limbic level (NPPO-CB) to manage responses conditioned by emotional states and mood and enhance integration between the autonomic and neuroendocrine nervous systems [6,7]; and finally, we employed the neuromuscular optimization (NMO) treatment to enhance the coordination and control of both agonist and antagonist muscles, both on the same side (ipsilateral) and across sides (heterolateral).

To assess the impact of these treatments, we employed manual muscle testing (MMT) [8], a well-established and widely accepted assessment tool. MMT enabled us to quantitatively evaluate muscle strength and functional limitations in individuals diagnosed with neuromuscular disorders, including those experiencing PPS.

## 2. Materials and Methods

### 2.1. Ethics

This study was approved by the Ethics Committee of the Federal University of São Paulo—UNIFESP, project CEP/UNIFESP n: 1343/2020, opinion number: 4,526,882. This study was conducted in accordance with the ethical guidelines of the Declaration of Helsinki. Informed consent was obtained from all the subjects involved in this study.

### 2.2. Study Design

Open-label study.

### 2.3. Research Locations

The investigation was undertaken at the Division of Neuromuscular Diseases within the Department of Neurology and Neurosurgery at the UNIFESP, located in São Paulo, Brazil.

### 2.4. Study Replicability

The REAC device (BENE mod 110—ASMED Srl Via Charta 77, 50018, Scandicci, Italy) used in this study ensures treatment consistency across administrations. Fixed parameters, set by the manufacturer, control the device during the administration of the treatments used. Operators cannot modify these parameters.

### 2.5. Population

#### 2.5.1. Sample Size Determination and Power Analysis

To ensure a statistically robust study, we performed a power analysis using G*Power software 3.1 [9]. This analysis considered a one-tailed Wilcoxon signed-rank test with a medium effect size (0.5), a 5% chance of a false positive (alpha), and a desired power of 50%. Based on these parameters, we determined we needed at least 13 participants. To account for potential dropouts, we decided to recruit additional participants beyond the minimum sample size. A total of 17 patients with PPS were enrolled in this study. All pre-existing treatments, pharmacological or non-pharmacological, were continued throughout the study period in accordance with their physicians’ recommendations.

#### 2.5.2. Inclusion and Exclusion Criteria

To be eligible for this study, the patients were required to meet the following criteria: confirmed diagnosis of PPS for more than one year; age between 18 and 65 years, regardless of gender; electroneuromyography test results compatible with poliomyelitis; ability to walk at least three meters with or without assistance and orthoses; preserved ability for verbal communication; ability to comprehend the study information and provide informed consent.

Patients who met any of the following criteria were excluded from this study: associated diseases that also lead to motor neuron involvement, such as amyotrophic lateral sclerosis (ALS) and acute motor axonal neuropathy (AMAN), diabetes mellitus, alcoholism, thyroid disease, multifocal conduction block, multiple sclerosis, nutritional deficiency, autoimmune diseases, or exogenous intoxication (heavy metals and insecticides).

### 2.6. Intervention and Study Stages

**Figure 1:**
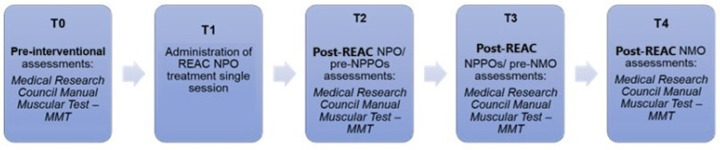
Study flowchart and phases.

Once patients had been recruited in accordance with the inclusion and exclusion criteria and informed consent had been obtained, the study commenced. The patients underwent four treatment protocols: neuro-postural optimization (NPO), neuro-psycho-physical optimization (NPPO), neuro-psycho-physical optimization—cervical brachial (NPPO-CB), and neuromuscular optimization (NMO), divided into different stages (Figure 1). It is crucial to emphasize that each treatment protocol was preceded and followed by efficacy assessment procedures. The protocol spanned a period of 12 weeks (about 3 months), during which evaluations and applications were conducted. All the assessments were performed before and after each stage of the research.

T0: Pre-intervention period: all the assessments were conducted at this time.

T1: NPO administration.

T2: Post-REAC NPO/pre-NPPO assessments: The NPPOs were administered concurrently, with patients receiving the NPPO initially, followed by the NPPO-CB, entailing 18 treatment cycles. The NPPO-CB was administered immediately following each NPPO application. This resulted in eight daily applications (4 NPPO + 4 NPPO-CB) separated by a one-hour interval between each.

T3: Post-REAC NPPOs and pre-NMO assessments.

T4: NMO administration. The treatment plan comprises 10 applications, divided into three cycles. During the initial two cycles, four applications were conducted daily, while in the third cycle, two applications were administered, with a one-hour interval between them.

### 2.7. Valuation Instruments

#### Medical Research Council Manual Muscular Test—MMT

This scale is typically employed for the clinical assessment of MS in patients with neuromuscular diseases. MMT, which was developed in 1943, was originally utilized to assess MS in patients with poliomyelitis. It comprises scores of 0–5 points for each muscle group tested. A score of 0 indicates no contraction, while a score of 1 indicates a visible contraction without movement of the segment. A score of 2 indicates active movement with the elimination of the action of gravity. A score of 3 indicates active movement with the action of gravity. A score of 4 indicates active movement with the action of gravity and resistance. A score of 5 indicates normal strength.

The following muscle groups were subjected to testing: flexors and extensors of the neck, trunk, shoulder, elbow, wrist, fingers, knee, ankle, and toes; abductors and adductors of the shoulder and hip. In all the strength tests, measurements were taken on the right and left sides in order to minimize the influence of laterality and to exclude asymmetric involvement of muscle groups.

The higher the score, the more successful the performance in the task. It should be noted that the instrument can be used regardless of the degree of functional impairment of the individual; for example, it can be used in ambulant or non-ambulant patients, as well as for the assessment of manual function.

After the MS assessment, the percentage of strength was computed using the Medical Research Council (MRC) index.

MRC Index: sum of score × 100% n 0 of muscles tested × 5.

The muscles were grouped in eighteen different ways to assess the distal and proximal components of MS in these patients, as listed below.

MS of Cervical muscles—(Cervical flexors + cervical extensors) × 100/(2 × 5);

MS of Trunk muscles—(Trunk flexors + trunk extensors + trunk rotators) × 100/(3 × 5);

MS of proximal muscles of the upper limbs (ULs)—(Right (R) and left (L) scapular elevators + shoulder flexors R and L + shoulder extensors R and L + shoulder abductors R and L + shoulder adductors R and L) × 100/(10 × 5);

MS of distal muscles of (ULs)—(Elbow flexors R and L + elbow extensors R and L + wrist flexors R and L + wrist extensors R and L + supinators R and L +pronators R and L + fingers flexors R and L + fingers extensors of fingers R and L + fingers adductors R and L + thumb opponents R and L + fifth finger abductors R and L) × 100/(22 × 5);

MS of proximal muscles of the lower limbs (LLs)—(Hip flexors R and L + hip extensors R and L + hip abductors R and L + hip adductors hip R and L) × 100/(8 × 5);

MS of distal muscles of the LL—(Knee flexors R and L + knee extensors R and L + dorsiflexors R and L + plantar flexors R and L + fingers flexors R and L + fingers extensors R and L + invertors R and L + evertors R and L + hallux flexors R and L + hallux extensors R and L) × 100/(20 × 5);

MS of the proximal muscles of the right upper limb (RUL)—(Scapula elevators R + shoulder flexors R + shoulder extensors R + abductors R + adductors) × 100/(5 × 5);

MS of the proximal muscles of the left upper limb (LUL)—(Scapular elevators L + shoulder flexors L + shoulder extensors L + abductors L + adductors L) × 100/(5 × 5);

MS of the proximal muscles of the right lower limb (RLL)—(Hip flexors R + hip extensors R + hip abductors R + hip adductors R) × 100/(4 × 5);

MS of the proximal muscles of the left lower limb (LLL)—(Hip flexors L + hip extensors L + hip abductors L + hip adductors L) × 100/(4 × 5);

MS of the distal muscles of RUL—(Elbow Flexors R + elbow extensors R + supinators R + pronators R + Wrist flexors R + wrist extensors R + fingers flexors R + finger extensors R + fingers adductors R + thumb opponents R + fifth finger abductors D) × 100/(11 × 5);

MS of the distal muscles of the LUL (elbow flexors L + elbow extensors L + supinators L + pronation L + wrist flexion L + wrist extension L + fingers flexion L + fingers extension E + fingers adduction L+ thumb opponents L + fifth finger abduction L) × 100/(11 × 5);

MS of the distal RLL muscles—(Knee flexors R + knee extensors R + dorsiflexors R + plantar flexors D + fingers flexors R + fingers extensors R + invertors R + evertors R + hallux flexors R + hallux extensors R) × 100/(10 × 5);

MS of the distal LLL muscles—(Knee flexors L + knee extensors L + dorsiflexors L + plantar flexors L + fingers flexors L + fingers extensors L invertors L + evertors L + hallux flexors L + hallux extensors L) × 100/(10 × 5);

MS of the proximal + distal muscles of RUL—(Scapula elevators R + shoulder flexors R + shoulder extensors R + abductors R + adductors R + elbow flexors R + elbow extensors R + wrist flexors R + wrist extensors R + supinators R + pronators R + fingers flexors R + fingers extensors R + adductors of fingers R + thumb opponents R + fifth finger abductors R) × 100/(16 × 5);

MS of the proximal + distal LUL muscles—(Scapula elevators L+ shoulder flexors L + shoulder extensors L + abductors L + adductors L + Elbow Flexors L + Elbow extensors L + wrist flexors L + wrist extensors L + supinators L + pronators L + fingers flexors L + fingers extensors L + finger adductors L + thumb opponents L + fifth finger abductors L) × 100/(16 × 5);

MS of the proximal + distal muscles of the RLL—(Hip flexors R + hip extensors R + hip abductors R + hip adductors R + Knee flexors R + knee extensors R + dorsiflexors R + plantiflexors R + fingers flexors R + fingers extensors R + invertors R + evertors R + hallux flexors R + hallux extensors D) × 100/(14 × 5);

MS of the proximal + distal LLL (Hip flexors L + hip extensors L + hip abductors L + hip adductors L + Knee flexors L + knee extensors L + dorsiflexors L + plantar flexors L + fingers flexors L + fingers extensors L + invertors L + evertors L + hallux flexors L + hallux extensors L) × 100/(14 × 5).

### 2.8. REAC Technology

REAC technology is a non-invasive, electro-biological approach that modulates cellular function through the emission of specific radio electric signals asymmetrically conveyed inside the body.

These signals interact with the cellular endogenous bioelectric field, leading to improved cellular communication, metabolic activity, and tissue repair [10].

The underlying mechanism of REAC technology involves the generation of an asymmetrical radio electric field, which induces a potential difference across the cell membrane, resulting in increased cellular activity. This field also triggers the flow of ions through the cell membrane, further enhancing the biological effects through interactions with the endogenous bioelectric field.

The REAC device (BENE 110, ASMED Srl, Scandicci, Florence, Italy) is capable of producing customized radio electric signals tailored to the individual’s physiological and clinical characteristics. REAC technology has been successfully applied in various medical fields, including pain management [11], neurological disorders, orthopedics, dermatology, wound healing, anti-senescence, and direct cell reprogramming.

Clinical trials have demonstrated the safety and tolerability of REAC neurobiological protocol treatments, with no significant adverse effects reported.

### 2.9. REAC Treatments

#### 2.9.1. REAC NPO

NPO is a therapeutic intervention that aims to modulate the electrometabolic activity of the nervous system and optimize postural control. This treatment is based on the principle that postural control is a complex process that involves not only the musculoskeletal system but also the integration of sensory information from multiple sources, such as vision, proprioception, and the vestibular system.

The REAC NPO treatment involves a single session lasting only a few milliseconds. During this session, a non-invasive asymmetric conveyer probe (ACP) is applied to a specific area of the auricle. The ACP conveys the radio electric field in the body and brain, inducing a series of neurophysiological responses in the nervous system by enhancing the function of the neurotransmission processes that are governed by endogenous bioelectrical activity [12].

These responses promote the progressive restoration of postural and neuro-psycho motor control for the patient, resulting in the immediate and stable disappearance of functional dysmetria (FD).

#### 2.9.2. REAC NPPO and NPPO-CB

The REAC NPPO and NPPO-CB treatments are non-invasive neurobiological modulation therapies designed to optimize the function of the nervous, psychological, and physical systems. These treatments involve the application of an ACP to specific points of the auricle (NPPO) or the cervicobrachial region (NPPO-CB) while the patient is in a supine or sitting position, if necessary.

The duration of the NPPO and NPPO-CB treatments is approximately three seconds and four minutes, respectively [6,7].

These therapies have demonstrated efficacy in addressing a range of conditions, including chronic pain [11], anxiety, depression [6,7], insomnia, fatigue, and cognitive impairment. Indeed, these treatments also rely on the modulation of endogenous bioelectrical activity to enhance neurotransmission processes, which underlies their mode of action [12].

#### 2.9.3. REAC NMO

NMO is a set of treatment protocols of REAC neuromodulation therapeutic protocols. These protocols are designed to optimize muscle function by restoring the equilibrium between agonist and antagonist muscles, both ipsilateral and contralateral, in pathological and dysfunctional conditions. This approach enables a gradual and progressive correction of neuro-psychomotor abnormalities, leading to the restoration of proper muscle and postural management.

Typically, multiple therapeutic cycles are implemented, tailored to the individual’s health status or specific medical condition. During NMO treatments, the targeted areas are covered with a specialized asymmetric conveyer probe (ACP) that is connected to the REAC BENE 110 device.

### 2.10. Statistical Analysis

IBM SPSS^®^ Statistics software, version 23, was employed for conducting the statistical procedures.

A repeated measures analysis of variance (ANOVA-RM) was conducted to evaluate the MRC index across four conditions: T0, T2, T3, and T4.

Additionally, post hoc analyses (Bonferroni’s post hoc test) were performed on the muscle groups that showed a *p* < 0.05 in the ANOVA-RM test.

## 3. Results

Seventeen patients diagnosed with PPS participated in this study. Of the total number of patients, 70.6% were female, with a mean age of 54.9 ± 4.5 years. The most prevalent residual sequelae of poliomyelitis were diparesis (52.9%) and monoparesis (41.2%). The most frequently affected limb was the LLL, occurring in 76.5% of the participants. At the outset of the study (T0), the participants exhibited a primary physical status of tetraparesis (58.8%) and paraparesis (41.2%). Table 1 illustrates a more pronounced degree of paresis in the LLL when the initial assessment was conducted (T0).

Table 2 shows the mean and standard deviation of the MRC index per body segment at different times of the study (T0–T4).

**Figure 2:**
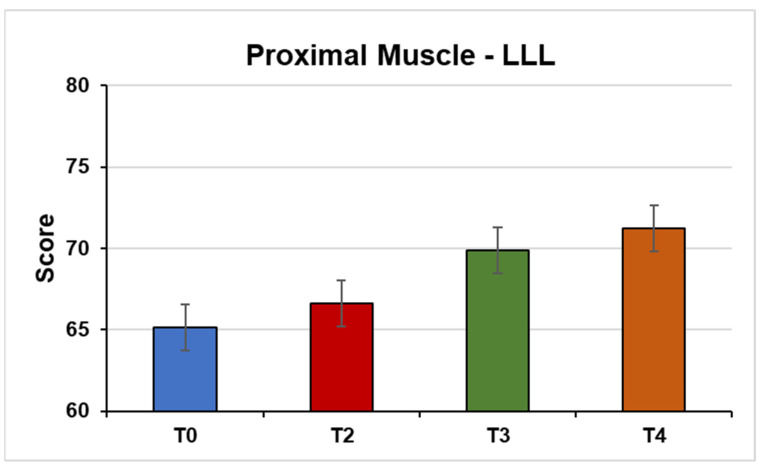
This figure shows the mean strength of the proximal muscles of the left lower limb from the MMT test under the conditions (T0) Pre-NPO (N = 17), (T2) NPPO (N = 17), (T3) Pre-NMO (N = 17), and (T4) Post-NMO (N = 16).

A repeated measures analysis of variance (ANOVA-RM) was conducted to evaluate the MRC index in four conditions: T0, T2, T3, and T4. The overall result of the ANOVA-RM showed statistically significant differences among the conditions described above in the following muscle groups:

Proximal muscles of the LLL (F (3, 45) = 6.54, *p* = 0.002; η^2^ = 0.30) (Figure 2).

**Figure 3:**
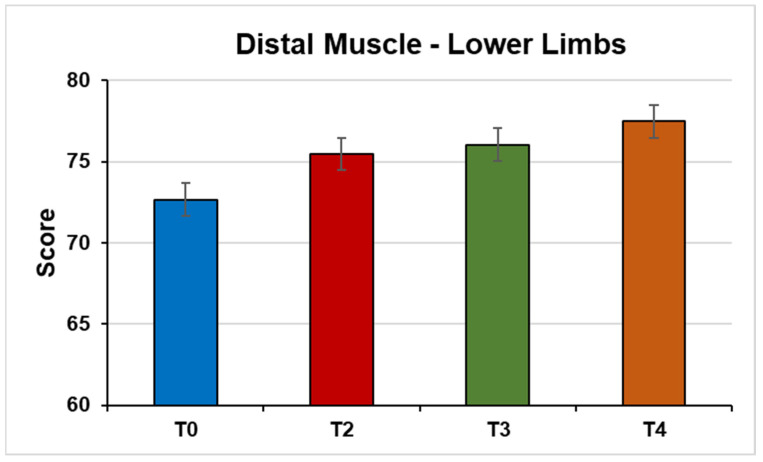
This figure shows the mean strength of the distal muscles of the lower limbs from the MMT test under the conditions (T0) Pre-NPO (N = 17), (T2) NPPO (N = 17), (T3) Pre-NMO (N = 17), and (T4) Post-NMO (N = 16).

Distal muscles of the LLs (F (3, 45) = 11.23, *p* < 0.0001; η^2^ = 0.42) (Figure 3).

**Figure 4:**
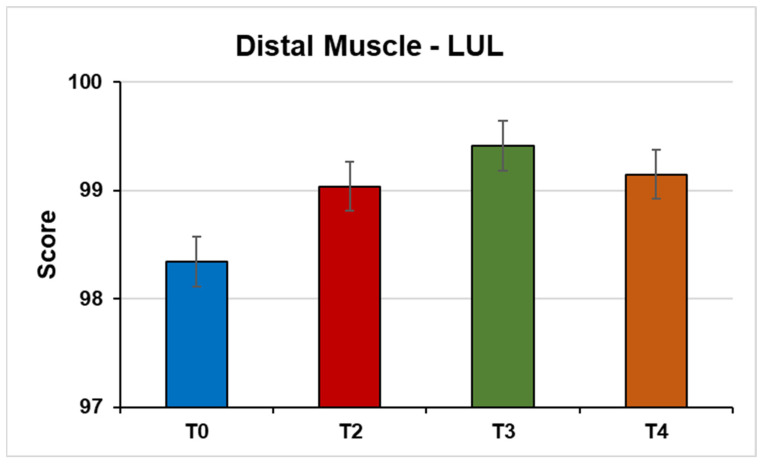
This figure shows the mean strength of the musculature distal MSE from the MMT test under the conditions (T0) Pre-NPO (N = 17), (T2) NPPO (N = 17), (T3) Pre-NMO (N = 17), and (T4) Post-NMO (N = 16).

Distal muscles of the LUL (F(1, 82, 27.35) = 4.55, *p* = 0.02; η^2^ = 0.23) (Figure 4).

**Figure 5:**
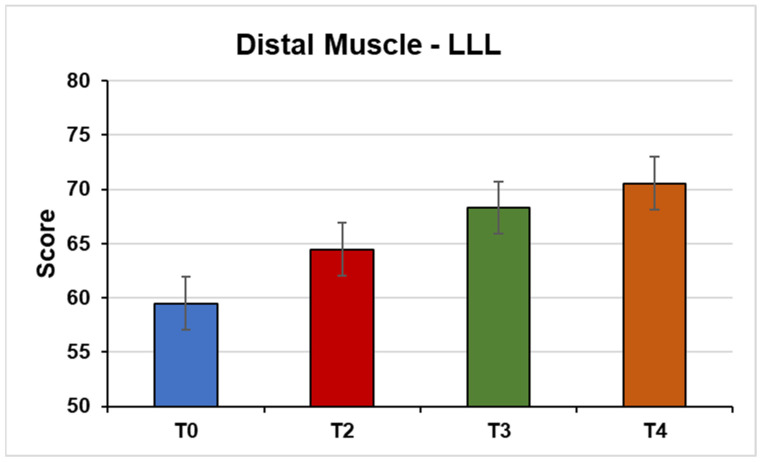
This figure shows the mean strength of the distal muscles of the left lower limb from the MMT test under the conditions (T0) Pre-NPO (N = 17), (T2) NPPO (N = 17), (T3) Pre-NMO (N = 17), and (T4) Post-NMO (N = 16).

Distal muscles of the LLL (F(1, 32, 19.88) = 5.51, *p* = 0.02; η^2^ = 0.26) (Figure 5).

**Figure 6:**
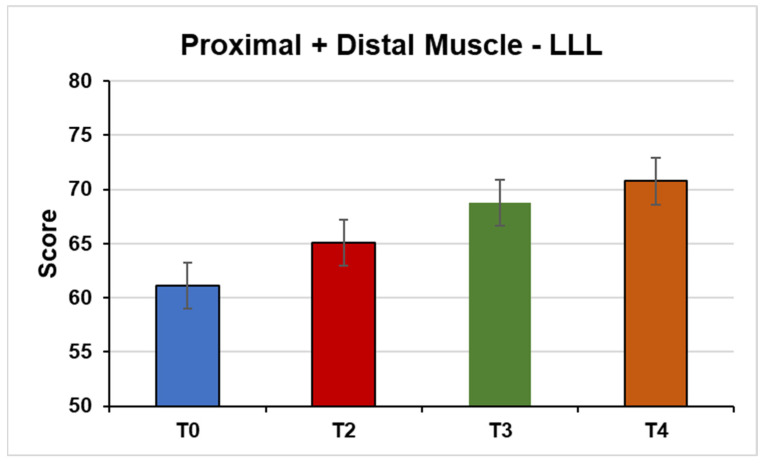
This figure shows the mean strength of the proximal + distal muscles of the left lower limb E from the MMT test under the conditions (T0) Pre-NPO (N = 17), (T2) NPPO (N = 17), (T3) Pre-NMO (N = 17), and (T4) Post-NMO (N = 16).

Proximal and distal muscles of the LLL (F(1, 33, 19.98) = 7.57, *p* = 0.01; η^2^ = 0.33) (Figure 6).

For the other muscle groups, the ANOVA-RM test generally showed that the conditions did not differ significantly, i.e., they had *p* > 0.05. These include the following: cervical muscles (F(1, 17, 17.55) = 1.81, *p* = 0.19; η^2^ = 0.10), trunk muscles (F(1, 48, 22.25) = 2.41, *p* = 0.12; η^2^ = 0.13), proximal muscles of the upper limbs (ULs) (F(1, 26, 18.95) = 0.52, *p* = 0.51; η^2^ = 0.03), distal muscles of the ULs (F(1, 87, 28.07) = 3.03, *p* = 0.06; η^2^ = 0.16), proximal muscles of the LLs (F(1, 44, 21.59) = 3.28, *p* = 0.07; η^2^ = 0.17), proximal muscles of the RUL (F(1, 15, 17.28) = 0.72, *p* = 0.42; η^2^ = 0.04), proximal muscles of the LUL (F(1, 36, 20.50) = 0.72, *p* = 0.44; η^2^ = 0.04), proximal muscles of the RUL (F(1, 24, 18.69) = 1.71, *p* = 0.20; η^2^ = 0.10), distal muscles of the RUL (F(1, 56, 23.43) = 0.62, *p* = 0.50; η^2^ = 0.04), distal muscles of the RLL (F(1, 10, 16.61) = 0.36, *p* = 0.57; η^2^ = 0.02), proximal and distal muscles of the RUL (F(1, 38, 20.83) = 1.18, *p* = 0.30; η^2^ = 0.07), proximal and distal muscles of the LLL (F(1, 91, 28.65) = 3.21, *p* = 0.06; η^2^ = 0.17), and proximal and distal muscles of the RLL (F(1, 38, 20.74) = 0.41, *p* = 0.59; η^2^ = 0.02).

Post hoc analyses (Bonferroni’s post hoc test) were performed on the muscle groups that had a *p* < 0.05 in the ANOVA-RM test. In this sense, the *p* values obtained in each comparison are represented in Table 3.

## 4. Discussion

PPS is a neurodegenerative condition that requires further investigation to identify effective therapies. Currently, the primary treatment for PPS is based on non-pharmacological interventions, lifestyle changes, physical therapy, training programs, and the prevention of secondary complications. Muscle weakness is considered one of the main symptoms of PPS. The present study aimed to assess the efficacy of REAC neurobiological optimization treatments in MS in individuals diagnosed with PPS. It was possible to verify the asymmetrical involvement of the patients, with the main sequelae of acute poliomyelitis being diparesis, with the LLL more affected. In order to objectively assess strength, MMT was employed in the present study, while the MRC index was utilized to more effectively group muscles in subsequent data analysis. In the initial assessment, it was observed that the participants predominantly exhibited quadriparesis, with the LLL being the most affected. Despite being the limb most affected and, in the overwhelming majority of cases, a limb that presents extensive muscular hypotrophy, the LLL muscle group demonstrated the most improvement following the implementation of the protocol. This is illustrated by a comparison of the initial assessment (T0) with the final assessment (T4), as evidenced in Table 2.

Upon examination of the research results, it becomes evident that the T2 findings indicate a notable improvement, particularly in the LLL distal muscle groups, following the administration of the NPO therapeutic protocol. The NPO protocol acts to counteract the entropic decline of connectomic functions by rebalancing and optimizing cellular endogenous bioelectrical activity within the intricate neural network. The optimization of endogenous bioelectrical activity results in improved and more functional brain electro-metabolic activity at the cellular level, which in turn triggers a reorchestration of the connectome. In turn, this improves functionality and optimizes neuronal functions dedicated to the execution of specific motor tasks, despite the presence of pre-existing physical damage. The reorganization of endogenous electrical fields appears to be responsible for these effects on the nervous system, as it is known that these fields are fundamental for synaptic communication, especially regarding motor control. These findings are consistent with those of a study of 50 individuals with Parkinson’s disease, which demonstrated that REAC treatments can improve strength, balance, and overall physical function. The mean time to complete the five-time sit-to-stand test was significantly reduced following the treatments.

An improvement in functionality has also been achieved in numerous other neurological diseases, including Parkinson’s disease, as well as PPS.

The research participants demonstrated improvements in MS throughout the entire protocol, with statistical significance observed in specific segments, as was the case with the distal LLL muscle group. Furthermore, motor improvement was also obtained when T3 was analyzed after the application of NPPO and NPPO-B, which are designed to improve mood and behavioral disorders. This interaction suggests that the neuropsychological state exerts a modulating influence on motor performance, even in complex conditions such as PPS.

Individuals who were afflicted with poliomyelitis during childhood have a history of experiencing stress from both a physical and psychological perspective. From a motor perspective, due to the significant asymmetry of involvement, whether in acute poliomyelitis or PPS, this population is subjected to a phase of constant adaptation, often requiring compensation that allows for the maintenance of functionality. However, this is achieved, in most cases, through an increase in energy expenditure. These adaptations will be influenced by several factors, including the individual’s clinical condition, socio-economic status, rehabilitation, surgeries, and lifelong occupation. A study was conducted with polio survivors who exhibited unilateral plantar flexor paresis. The results demonstrated that the energy cost of walking was 24% higher than the reported reference value [13]. The reduction in gait stability was due to variability in the step width and length of the affected leg and the stability margin of the unaffected leg, which were identified as detrimental factors for this increase in energy cost [13].

The results of applying the REAC NMO (T4) neuromodulation protocol, such as improvement in MS, particularly in distal muscles, suggest a decrease in the dysfunctional adaptive component that negatively exacerbated the damage induced by poliovirus infection. This scenario illustrates how the disease can induce or exacerbate deterioration in motor strategies, resulting in dysfunction that is often indistinguishable from the primary outcome of the injury. The protocol functions by rebalancing motor coordination inconsistencies between agonist and antagonist muscles.

Corroborating this, a study carried out by this research team demonstrated an improvement in handgrip strength, mobility, functional performance, and fatigue in individuals with PSS who underwent the four REAC treatment protocols (NPO, NPPO+NPPO-CB, and NMO), evidencing the use of REAC technology as a new treatment strategy to be used with this population.

The treatments utilized in this study demonstrated superior efficacy in more compromised muscles, supporting the hypothesis of enhanced motor organization and strategy improvement.

This finding aligns with previous studies employing functional magnetic resonance imaging (fMRI) examinations.

**Figure 7:**
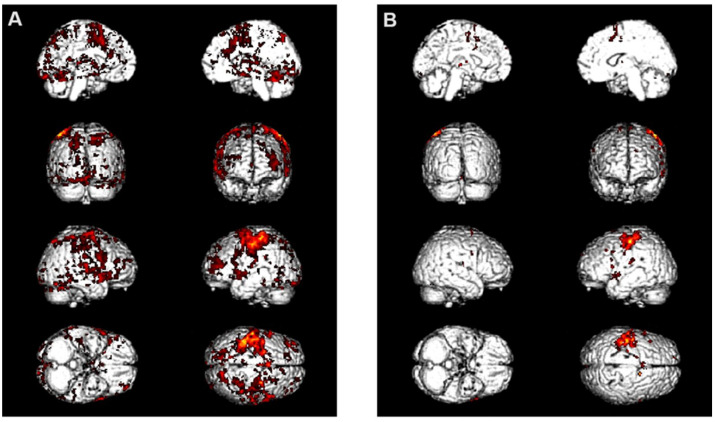
This figure shows the effects of the REAC NPO as a significantly reduced signal amplitude following a motor task (**B**) compared to that observed prior to the REAC NPO (**A**).

An fMRI example of the enhanced motor organization and strategy improvement in patients with neurodegenerative disease is shown in Figure 7A,B.

Figure 7A illustrates a fMRI of a right-handed male subject with multiple sclerosis performing a finger-tapping task before receiving the REAC NPO treatment. Figure 7B shows the same subject one hour after receiving the REAC NPO treatment (see Appendix A). The disappearance of large activation clusters in motor and non-motor areas not specifically involved in the motor task execution was observed, which were present before the treatment (Figure 7A). Figure 7B shows that activation is specifically localized to motor areas directly involved in the execution of the motor task. What is highlighted in Figure 7B allows us to better understand the functional optimization mechanism underlying the results described in this study.

Based on the principle and objective of optimizing neurofunctional outcomes through the REAC protocols employed in this study, it is hypothesized that these REAC therapeutic protocols exert a heightened impact on the less functional limb. Given that PPS is a degenerative and progressive disease affecting all muscles, enhancing functionality in the most affected limb could potentially mitigate overuse of the unaffected limb.

The lack of statistically significant improvement in other muscle groups can be attributed to the relatively lower incidence of motor dysfunction in these muscle groups, where the dysfunction was observed to be less pronounced.

The advent of novel therapeutic approaches for this demographic group offers the potential to reframe the evaluation of existing perspectives and promote a sense of optimism regarding the possibility of enhancing the quality of life of those affected, thereby reducing the impact of their daily difficulties. The findings indicate that the REAC neurobiological optimization treatments may be a promising approach for the management of PPS, particularly in terms of increasing muscle strength in specific muscle groups.

## 5. Conclusions

In conclusion, this study provides evidence suggesting that REAC NPO, NPPO, NPPO-CB, and NMO treatments may be beneficial for improving MS in individuals with PPS. The results demonstrate a notable improvement in the muscles most affected by polio (LLL). This suggests that the intervention may be an effective aid in reorganizing the circuits of motor patterns, as well as possibly reducing the overload of the most functional limb.

## Figures and Tables

**Table 1:** Initial involvement of participants (MMT).

Upper Limbs		
	**RUL**	LUL
**Proximal**	99.4 ± 1.2	99.5 ± 1.9
**Distal**	98.8 ± 1.6	98.3 ± 2.2
**Lower Limbs**		
	RLL	LLL
**Proximal**	69.4 ± 30.2	65.1 ± 21.6
**Distal**	85.8 ± 24.5	59.5 ± 28.7

Legend: RUL—right upper limb; LUL—left upper limb; RLL—right lower limb; LLL—left lower limb.

**Table 2:** Assessment of muscular strength distributed by segments (proximal + distal limb muscles) presented using the MRC index.

	T0	T2	T3	T4
**RUL**	99 ± 1.2	99.3 ± 1.1	99.6 ± 0.9	98.8 ± 2.1
**LUL**	98.7 ± 2	99.1 ± 1.7	99.4 ± 1.3	99.3 ± 1.4
**RLL**	81.1 ± 24.6	81.8 ± 23.4	80.6 ± 28.3	82.5 ± 27.7
**LLL**	61.1 ± 22.8	65.1 ± 23.1	68.7 ± 23.4	70.8 ± 23.4

Legend: RUL—right upper limb; LUL—left upper limb; RLL—right lower limb; LLL—left lower limb.

**Table 3:** Results of the a posteriori comparison between the conditions in the muscle groups of the MMT test from the repeated measures analysis of variance.

Proximal + Distal Muscles of LLL	AVERAGE ± SD	T0	T2	T3	T4
**T0**	65.00 ± 22.24	-			
**T2**	66.40 ± 22.49	1.00	-		
**T3**	69.84 ± 20.48	0.12	0.17	-	
**T4**	71.25 ± 18.48	0.02	0.08	1.00	-
**Distal Muscles of LLs**	AVERAGE ± SD	T0	T2	T3	T4
**T0**	71.90 ± 19.56	-			
**T2**	74.68 ± 20.03	0.02	-		
**T3**	75.28 ± 20.86	0.04	1.00	-	
**T4**	77.46 ± 18.91	0.01	0.02	0.13	-
**Proximal+ Distal of LUL**	AVERAGE ± SD	T0	T2	T3	T4
**T0**	98.53 ± 2.14	-			
**T2**	99.04 ± 1.57	0.20	-		
**T3**	99.38 ± 1.02	0.14	0.65	-	
**T4**	99.15 ± 1.48	0.18	1.00	1.00	-
**Distal Muscles of LLL**	AVERAGE ± SD	T0	T2	T3	T4
**T0**	58.50 ± 29.35	-			
**T2**	63.43 ± 29.42	0.08	-		
**T3**	67.56 ± 30.80	0.17	1.00	-	
**T4**	70.56 ± 29.93	0.04	0.39	0.06	-
**Proximal+ Distal Muscles of LLL**	AVERAGE ± SD	T0	T2	T3	T4
**T0**	60.36 ± 23.37	-			
**T2**	64.28 ± 23.66	0.04	-		
**T3**	68.73 ± 23.39	0.07	0.89	-	
**T4**	70.75 ± 23.40	0.02	0.16	0.09	-

## Data Availability

Data are contained within the article.

## Appendix A

### Supplementary Information Regarding the Acquisition and Analysis of the fMRI Data Shown in Figure 7A,B

In this case, the fMRI examination was performed using an NT Intera 1.5T MRI scanner (Philips, Amsterdam, The Netherlands) to evaluate the cerebral areas involved during a simple motor task (finger tapping) (Figure 7A) before and immediately after the REAC NPO (Figure 7B). The area of interest was visualized using a survey protocol with images obtained along the axial, sagittal, and coronal planes. A volumetric T1-weighted gradient echo sequence (T1 three-dimensional turbo field echo, repetition time = 13 ms, echo time = 3 ms, flip angle = 30°) and an fMRI sequence (T2 echo planar imaging fast-field echo, repetition time = 3000 ms, echo time = 50 ms, flip angle = 90°) were employed. Before administering REAC NPO, the subjects were placed in a supine position and instructed to perform a 30 s task of self-paced right finger-to-right thumb opposition movements, alternating with equal periods of rest. During the fMRI acquisition, the subjects completed 96 dynamic sequences divided into blocks of ten (30 s each), with the first six sequences during rest being discarded. This fMRI examination was repeated an hour later. The fMRI data were analyzed using Statistical Parametric Mapping 2 (SPM2; The Welcome Trust Center for Neuroimaging, London, UK) software, employing the familywise error rate correction routine to automatically set the correct value of *p* = 0.05 at the voxel level.
